# Maternal and cord blood adiponectin levels in relation to post-natal body size in infants in the first year of life: a prospective study

**DOI:** 10.1186/s12884-016-0978-9

**Published:** 2016-07-27

**Authors:** Zhe-qing Zhang, Qing-gui Lu, Jie Huang, Chang-ya Jiao, Shao-ming Huang, Li-mei Mao

**Affiliations:** Department of Nutrition and Food Hygiene, Guangdong Provincial Key Laboratory of Tropical Disease Research, School of Public Health, Southern Medical University, Guangzhou, 510515 People’s Republic of China

**Keywords:** Adiponectin, Cord blood, Infant, Growth, Prospective study

## Abstract

**Background:**

Adiponectin is an adipocyte hormone involved in energy homeostasis and metabolism. However, its role in early infancy is poorly understood.

**Methods:**

We recruited a total of 443 pregnant women and their children in this prospective study. Cord blood samples were successfully obtained from 331 neonates. Maternal and umbilical blood serum adiponectin were measured. The weight-, height- and BMI-for-age Z scores of infants at birth and at 3, 6 and 12 months of age were assessed.

**Results:**

Multiple linear regression analysis indicated that cord blood but not maternal serum adiponectin was positively associated with all of the anthropometric measures at birth (*P* < 0.01). Using Generalized Estimating Equation model after adjustment for sex, time, maternal age, gestational age, prepregnancy BMI, weight gain during pregnancy, maternal education, parity, history of miscarriage and mode of delivery, for every 1-μg/ml increment of maternal serum adiponectin, the height-for-age Z score during the first year of life increased by 0.026 (*P* =0.013) on average, and the height-for-age Z score of infants in the highest quartile of maternal serum adiponectin was 0.270 (95 % CI: 0.013–0.527) higher than those in the lowest quartile. The changes in weight-for-age Z score from birth decreased by 0.67 × 10^−2^ on average with every 1-μg/ml additional increase of cord blood adiponectin (*P* = 0.047). The infants in the highest quartile of cord blood adiponectin showed a −0.368 (95 % CI, −0.701–−0.035) decrease in weight-for-age Z score change from birth compared with those in the lowest quartile.

**Conclusions:**

Cord blood adiponectin concentration is a determinant of infant birth size and weight gain in the first year of life. Circulating maternal adiponectin during pregnancy may predict postnatal height growth.

**Electronic supplementary material:**

The online version of this article (doi:10.1186/s12884-016-0978-9) contains supplementary material, which is available to authorized users.

## Background

Obesity has received considerable attention as a major health threat with several etiologic influences [1]. Observational evidence suggests that a high birth weight and accelerated growth in infancy are associated with an increased risk of obesity in childhood and adulthood [2, 3]. Accumulating evidence indicates that the development of obesity and its comorbidities may be influenced by intrauterine factors [4].

Adiponectin is an adipokine that is secreted by adipose tissue and correlates inversely with obesity in adults [5]. It is also abundantly present in the cord blood of term neonates, at concentrations two to three times higher than those reported in adults [6]. However, studies to date have yielded markedly conflicting results on the relationship between maternal serum/cord blood adiponectin concentration and infant birth weight, ranging from an inverse association to no correlation to even a positive relationship [7–9]. Relatively few studies have evaluated the longer-term relationships of adiponectin level with growth [8, 10]. Different ethnicities show varied levels of adiponectin [11], and the effect of pharmacologic agents that work by altering adiponectin levels also differs by ethnic group [12]. Studies in adults have established that ethnicity modifies the relationship between adiponectin and obesity, which may be partly due to differences in fat distribution particularly in the visceral compartment, the capacity for fat storage, genetic background and differential exposure to important environmental influences [12–14]. It is possible that ethnicity alters the relationship between adiponectin concentration and infant growth. Therefore, what applies to other ethnic populations might not apply to the Chinese population. However, up to now, no studies have been conducted to evaluate the effects of maternal and umbilical blood adiponectin levels on the postnatal growth of children in China. Additionally, most existing studies have estimated differences in adiponectin circulation associated with a change in anthropometric measures between two time points only, rather than with trajectories estimated from multiple measures of growth.

The primary aim of this study was to determine whether maternal and cord blood adiponectin levels are related to the growth of children in early infancy in China.

## Subjects and Methods

### Subjects

The subjects were 443 pregnancy women aged 20–35 and their babies from a prospective cohort study in Guangzhou, China. They were recruited from the Maternal and Children’s Hospital of BaiYun and Yuexiu District, Guangzhou between September 2010 and November 2011. All of the women had delivered normal single infants at full term. We excluded those with multiple births, a history of diabetes, chronic hypertension, endocrine disorders and /or other severe maternal illnesses. A detailed explanation of the study purposes, procedure and requirements was given to the subjects. Written informed consent was obtained from all the subjects before enrolment. The study protocol was approved by the research ethics board of Tongji Medical College, China.

### Data collection

#### Clinical and demographic data

An interviewer-administered questionnaire was used to collect the demographic and historical information of the subjects, including their educational status, household income, personal medical and obstetrical history, information about their current pregnancy (including the delivery type, gestational duration, gender of the baby, the weight gain during pregnancy, and maternal pre-pregnancy weight) and height.

### Anthropometric measurements

After delivery, maternal height was measured to the nearest 0.1 cm and weight to the nearest 0.5 kg while the subjects were wearing light clothing and no shoes. Measurements of the infants’ weight (±0.1 kg) and length (±0.1 cm) were made at birth and at 3, 6, and 12 months of age (+/− 15 days) according to standardised techniques by using an infant stadiometer (length board) and infant digital scale. Body mass index (BMI) was calculated as weight (kilogram)/height in meters square.

### Adiponectin concentrations

Venous blood samples of mothers were obtained in the morning after overnight fasting when they were admitted to the hospital awaiting delivery. A total of 331 cord blood samples were successfully collected from the umbilical vein after the delivery of the baby and before the delivery of the placenta. All of the blood samples were placed on ice packs, stored in styrofoam containers, and returned to our laboratory. On arrival, the blood samples were centrifuged and aliquoted, and frozen at −80 °C until analysis. The adiponectin in the serum was determined by a commercially available ELISA (R&D Systems, Wiesbaden, Germany). The intra- and interassay coefficients of variation were 5.4 and 7.0 %, respectively.

### Statistical analysis

All analyses were conducted using SPSS 21.0 for Windows (SPSS, Inc., Chicago, USA). *P* < 0.05 was considered significant. All continuous variables were expressed as mean ± standard deviation, or median with inter-quartile range. They were checked for normality using the Kolmogorov–Smirnoff test. Categorical variables are presented as percentages.

The age- and sex-specific weight-, height- and BMI-for age Z-scores were generated based on the WHO standards [15]. Two multiple linear regression models using the enter method were applied to examine whether the correlations between the adiponectin level and body size measurements at birth were independent after adjustment for other potential covariates. In model 1, we adjusted for sex. And in model 2, we further adjusted for maternal age, gestational age, prepregnancy BMI, weight gain during pregnancy, maternal education, parity, history of miscarriage and mode of delivery. We did not log transform adiponectin since its residual plot did not indicate any violations of the linear regression model assumptions. The Generalized Estimating Equation (GEE) method with unstructured correlation matrix was used to estimate the relationship between the maternal and cord blood adiponectin levels and the weight-, height- and BMI-for age Z-scores at 3, 6 and 12 months of age, and their average changes from birth. This technique is applied to the analysis of longitudinal data that are an extension of generalised linear models, and was specially developed to account for autocorrelation due to serial measurements [16]. Analyses of the relationship between maternal/cord blood adiponectin concentration and anthropometric indicators and their changes at 3, 6 and 12 months of age were serially adjusted for confounding factors in two generalized estimating equation models: that is, a model adjusted for sex and time only; and a model adjusted for maternal age, gestational age, prepregnancy BMI, weight gain during pregnancy, maternal education, parity, history of miscarriage and mode of delivery in addition to sex and time. The adiponectin level was entered as either a continuous or a four-level categorical variable.

## Results

### Selected characteristics of the study subjects

Table 1 summarizes the characteristics of the study population and neonatal outcomes. The mean age of the study participants was 27.3 years and the median prepregnancy BMI was 19.8 kg/m^2^. The median increase in weight at the end of pregnancy was 15.0 kg. Fifty-two percent of the neonates were male. The median neonatal gestational age was 39.0 weeks. The adiponectin levels were significantly higher in the cord blood than in maternal serum (39.6 μg/ml vs. 6.9 μg/ml, *P* < 0.05). After adjustment for gestational age, weight gain during pregnancy and age, a weak positive correlation (*r* = 0.172, *P* = 0.004) was observed in adiponectin levels in maternal vs. fetal circulation. The anthropometric parameters at each visit are showed in Additional file 1: Table S1. In general, the weight-, height- and BMI-for-age Z scores at birth were lower than the reference but higher at 3, 6 and 12 months (Additional file 1: Figure S1).

**Table 1:** Characteristics of the subjects

Variables	Mean ± SD/Median (IQR)
Maternal variables	
Age at enrolment (year)	27.3 ± 4.2
Pregnancy BMI (kg/m^2^)	19.8 (2.9)
Weight gain during pregnancy (kg)	15.0 (6.0)
Caesarean section (%)	47.9
Education (%)	
Junior high school or less	41.2
Senior middle school	32.4
High school or above	26.6
Parity (%)	
1	61.7
2	28.4
3	9.9
History of miscarriage (%)	
0	84.4
1	11.1
≥ 2	4.5
Maternal serum adiponectin (μg/ml)	6.9 (5.3)
Newborn/infant variables	
Male (%)	52.2
Length of gestation (weeks)	39 (2)
Birth weight (g)	3250 (480)
Birth length (cm)	50.0 (1.0)
Birth BMI (kg/m^2^)	13.1 ± 1.2
Cord adiponectin (μg/ml)	39.6 (20.1)

*BMI*: body mass index; *SD*: standard deviation; *IQR*: inter-quartile range

### Adiponectin level and neonatal weight-, height- and BMI-for-age Z score

There were significant inverse associations between cord blood serum adiponectin level and the neonatal weight-, height- and BMI-for age Z scores (Table 2). After controlling for sex, maternal age, gestation age, prepregnancy BMI, weight gain during pregnancy, maternal education, parity, history of miscarriage and mode of delivery, for every 1-μg/ml increase in cord blood adiponectin, the weight-for-age Z score at birth increased by 1.00 × 10^-2^ (*P* < 0.0001). Equivalent estimations of height- and BMI-for-age Z score at birth were 0.69 × 10^-2^ (*P* = 0.010) and 1.02 × 10^-2^ (*P* = 0.002), respectively. No significant relationships were detected between maternal serum adiponectin circulation and fetal anthropometrics.

**Table 2:** Multiple linear regression analysis of the associations between maternal and cord blood adiponectin levels and weight-, height-, and BMI-for-age z scores at birth

	Maternal blood APN			Cord blood APN		
	β (×10^−2^)	SE (×10^−2^)	*P*	β (×10^−2^)	SE (×10^−2^)	*P*
Weight-for-age Z score						
Model 1	−1.27	0.91	0.162	1.33	0.28	<0.0001
Model 2	−0.41	0.79	0.610	1.00	0.25	<0.0001
Height- for-age Z score						
Model 1	−0.27	0.87	0.755	1.00	0.27	<0.001
Model 2	0.44	0.84	0.598	0.69	0.27	0.010
BMI-for-age Z score						
Model 1	−1.72	1.10	0.117	1.25	0.33	<0.001
Model 2	−0.81	1.00	0.419	1.02	0.32	0.002

Model 1: Adjusted by sex

Model 2: Adjusted by sex, maternal age, gestation age, prepregnancy BMI, weight gain in pregnancy preceding, maternal education, parity, history of miscarriage and mode of delivery, Method: entered

### Adiponectin level and anthropometric measures and their changes after birth

The GEE analysis of the associations between maternal and cord blood adiponectin and the weight-, height- and BMI-for age Z scores and their changes at 3, 6 and 12 months are shown in Table 3. After controlling for sex, time, maternal age, gestation age, prepregnancy BMI, weight gain during pregnancy, maternal education, parity, history of miscarriage and mode of delivery, for every 1-μg/ml additional increase of maternal blood adiponectin, the height-for-age Z score increased by 2.59 × 10^−2^ on average during the follow-up period (*P* = 0.013). And the changes in weight-for-age Z score decreased by 0.67 × 10^−2^ with every 1-μg/ml increment of cord blood adiponectin (*P* = 0.047).

**Table 3:** GEE analysis of the associations between maternal and cord blood adiponectin levels and the anthropometric parameters and their changes from birth during the first year of life

	Maternal blood APN			Cord blood APN		
	β (×10^−2^)	SE (×10^−2^)	*P*	β (×10^−2^)	SE (×10^−2^)	*P*
Weight-for-age Z score						
Model 1	1.35	0.83	0.107	0.63	0.29	0.030
Model 2	1.63	0.81	0.044	0.36	0.28	0.199
Height-for-age Z score						
Model 1	2.48	1.04	0.017	0.52	0.32	0.105
Model 2	2.59	1.05	0.013	0.19	0.32	0.560
BMI-for-age Z score						
Model 1	−0.27	0.72	0.709	0.59	0.28	0.034
Model 2	0.33	0.70	0.641	0.44	0.27	0.110
Changes in weight-for-age Z score						
Model 1	2.62	1.02	0.010	−0.68	0.35	0.048
Model 2	1.92	0.99	0.051	−0.67	0.34	0.047
Changes in height-for-age Z score						
Model 1	2.77	1.28	0.030	−0.55	0.34	0.099
Model 2	2.09	1.29	0.105	−0.50	0.33	0.120
Changes in BMI-for-age Z score						
Model 1	1.78	1.07	0.090	−0.65	0.41	0.112
Model 2	1.19	1.02	0.244	−0.62	0.41	0.128

Model 1: Adjusted by sex and time

Model 2: Adjusted by sex, time, maternal age, gestation age, prepregnancy BMI, weight gain in pregnancy, maternal education, parity, history of miscarriage and mode of delivery

**Fig. 1:**
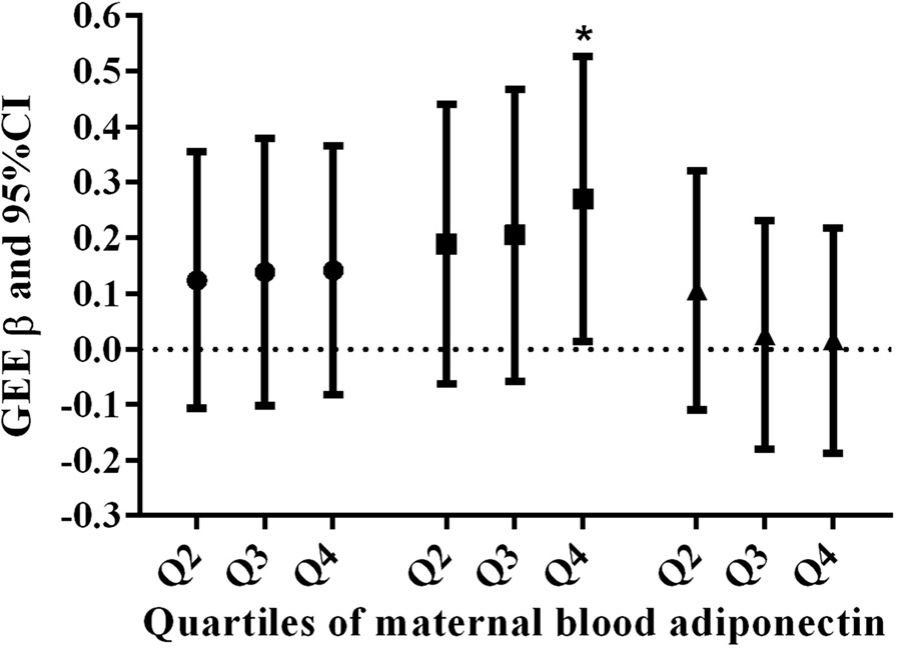
GEE analysis of the relationship between quartiles of maternal serum adiponectin and body-size Z score during the first year of life after adjustment for sex, time, maternal age, gestation age, prepregnancy BMI, weight gain during pregnancy, maternal education, parity, history of miscarriage and mode of delivery. Weight-for-age Z score: ●; Height-for-age Z score: ■; BMI-for-age Z score: ▲; *: *P* < 0.05. β values represent difference in body size Z score with the reference category, i.e., lowest quartile of adiponectin

**Fig. 2:**
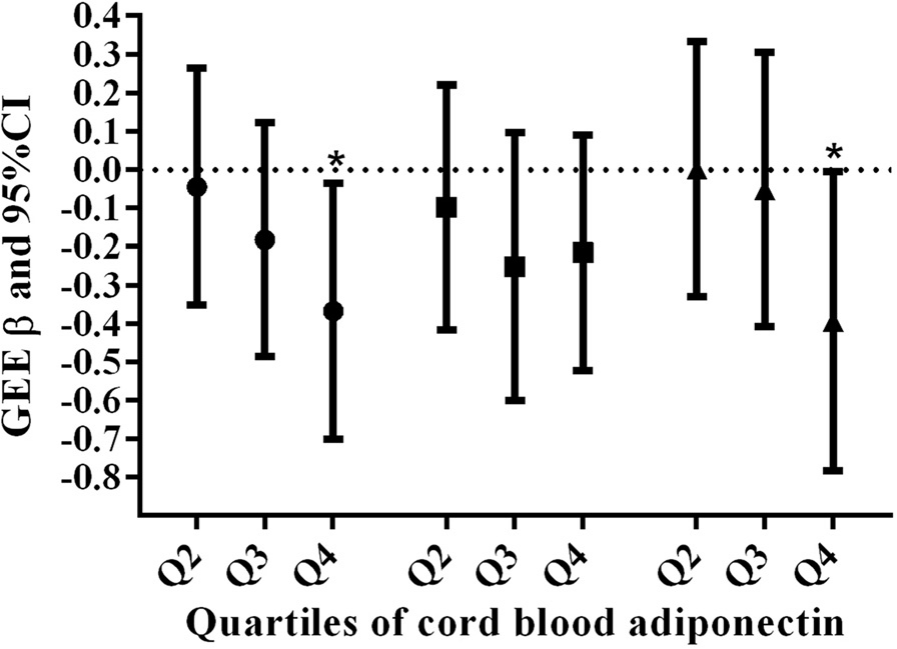
GEE analysis of the relationship between quartiles of cord blood adiponectin and body-size Z score changes from birth during the first year of life after adjustment for sex, time, maternal age, gestation age, prepregnancy BMI, weight gain in pregnancy preceding, maternal education, parity, history of miscarriage and mode of delivery. Weight-for-age Z score: ●; Height-for-age Z score: ■; BMI-for-age Z score: ▲; ^*^: *P* < 0.05. β values represent difference in body size Z score change with the reference category, i.e., lowest quartile of adiponectin

When the maternal blood serum adiponectin levels were ranked in quartiles, the height-for-age Z score of infants in the highest quartile of maternal serum adiponectin was 0.270 (95 % CI: 0.013–0.527) higher than that in the lowest quartile (Fig. 1) after adjustment for covariates as mentioned above. Figure 2 demonstrates the associations between the quartiles for cord blood serum adiponectin and changes in the anthropometrics Z score. Infants in the highest quartile of cord blood adiponectin showed a −0.368 (95 % CI, −0.701–−0.035) decrease in weight-for-age Z score change and a −0.395 (−0.784–−0.006) decrease in BMI-for-age Z score compared with the lowest quartile. No differences were found in the changes in body size Z scores from birth among the quartiles for maternal serum adiponectin or in the body size Z scores themselves among the quartiles of cord blood adiponectin (data not shown).

## Discussion

This prospective study is the first to report the influence of maternal and cord blood adiponectin levels on neonatal weight-, height- and BMI-for-age Z score at birth and their changes at 3, 6 and 12 months of age. The main finding of our study is that cord blood adiponectin was positively associated with anthropometric measures at birth but inversely correlated with weight-for-age Z score changes from baseline during the first year of life after adjustment for potential covariates. Maternal blood adiponectin concentrations were significantly lower than those of the umbilical blood and showed a positive relationship with height-for-age Z score during the first year of life.

We confirm data from previous observational studies showing that cord blood adiponectin levels were several folds higher than those seen in adults [17, 18]. The recent study of Luo et al. observed a statistically significant positive correlation in adiponectin levels in maternal and fetal circulation (*r* = 0.3, *P* < 0.0001) [19]. A significant positive correlation in adiponectin levels in maternal vs. fetal circulation adjusted by covariates was also detected in our data (*r* = 0.172, *P* = 0.004). Genetic variation in the adiponectin gene or its regulating elements may be responsible for this maternal-fetal correlation. In contrast, only nonstatistically significant positive correlations have been reported in most previous studies [6, 20, 21]. Insufficient study power in some studies (*n* = 51–74) [20, 21] and differences in the adjustment for potential confounders may partly explain the null findings in previous studies.

The positive correlation between cord blood adiponectin and fetal anthropometrics in our study is in agreement with some studies [6, 8, 21] but not all [22, 23]. Several factors may explain the lack of consistency in this regard. In the study of Lindsay et al., the absence of a significant association may be explained by the confounding effect of the existence of pre-disease states in some of the individuals included in the populations that were studied [9]. The other studies were limited by a small sample size (44–74), and thus they may have missed associations due to a lack of statistical power [20, 22, 23]. Additionally, studies in adults have indicated that the correlations between adiponectin and adiposity are modulated by ethnicities [12]. Whether ancestral background also accounts for disparities in the relationship between adiponectin and obesity in neonates needs to be further elucidated.

A few prospective studies have been conducted on the role of cord and maternal blood adiponectin levels in the postnatal growth of infants. In a sample of 588 children participating in the prospective prebirth cohort study Project Viva, Mantzoros et al. reported that cord blood adiponectin was inversely correlated with change in weight-for-length Z score (*r* = −0.12; *P* = 0.05) and weight-for-age Z score (*r* = −0.10; *P* = 0.04) from birth to 6 months but was not associated with change in length-for-age Z score (*r* = 0.03; *P* = 0.61) [10]. In agreement with the findings of Mantzoros et al. [10], we also found that neonates with a higher cord blood adiponectin gained less weight during the first year of life.

Most previous epidemiologic studies investigating the links between adiponectin levels and obesity in adults and children have demonstrated a negative relationship [24]. In fetal tissue, adiponectin is secreted not only by adipocytes, but also muscle and vascular cells, whereas in adult humans it is exclusively secreted by adipose tissue [25]. Fetal adipose tissue is also composed mainly of small newly differentiated adipocytes that lack the factors that are responsible for the inhibition of adiponectin production. Several lines of evidence suggest that senescent cells could accumulate in fat tissue with chronological aging and that these cells might contribute to age-related fat tissue inflammation and dysfunction [26]. Additionally, the available data suggest that brown adipose tissue (BAT) is more prevalent in children than in adults [27]. It is under strikingly different hormonal regulation than in white adipose tissue [28]. Moreover, adiponectin secretion from omental but not from sc adipocytes was negatively associated with measures of adiposity [29, 30]. The different origination of adiponectin, a decreasing sc adipose tissue/visceral adipose tissue ratio, the atrophy of brown adipose tissue and metabolic function changes in adipocytes with increasing age may in part explain the switch from a positive correlation between adiponectin and weight at birth to a negative correlation later in life [21, 28, 31]. In the Nurses’ Health Study, elevated adiponectin levels at baseline were associated with greater weight gain in healthy women [32], which is contrary to our findings in infants. The authors of the Nurses’ Health Study hypothesized that elevated adiponectin levels in humans was a sign of healthy adipose tissue and its capacity to adapt to more fat accumulation [32]. However, cellular stress and adipocyte overutilization with ageing led to metabolic dysfunction [26]. The variation in the metabolic function of adipocytes such as fat storage, the secretion and response to the modulation of adiponectin may collectively contribute to the discrepancies between infants and adults. In addition, BAT is especially abundant during infancy and its activation protects against weight gain [33]. Additional studies are required to determine whether BAT modulates weight changes by altering the secretion of adiponectin. Furthermore, some evidence suggested that adiponectin could reduce food intake and increase energy expenditure through action on the hypothalamus, which also provided a potential explanation for the negative association between cord adiponectin and weight gain [34].

The association between maternal adiponectin concentration and fetal growth are less clear. A negative correlation with birth weight has been reported by some studies [7], but not others [35, 36]. In this study, maternal adiponectin level was not a determinant of fetal size at birth, but was positively correlated with the height-for-age Z score during the first year after adjustment for cofounders. In theory, maternal adiponectin is unable to pass through the placental barrier due to its large molecular weight. However, Aye et al. [37] and Rosario et al. [38] have reported that adiponectin infusion in pregnant mice downregulates placental amino acid transporter activity and expression and decreases fetal growth. Thus, it is possible that maternal adiponectin circulation potentially modulates placental function, which, in turn, may affect the intrauterine environment of the foetus and the postnatal growth of infants. Further human studies are warranted to verify this hypothesis.

The strength of our study is its prospective design and repeated measures of anthropometric parameters in infants. We are the first to report the associations between adiponectin concentrations in maternal and umbilical blood and infant growth in early infancy in a Chinese population. However, our study has some limitations. First, not all of the subjects consented to the assessment of umbilical adiponectin status; thus, the results may be subject to potential bias. Second, the results may not be easy to generalize to other ethnic groups. Third, similar to all other previous studies, we did not determine fat mass directly, and did not measure the centrality of fat distribution. Thus, it remains unknown whether there is a relationship between adiponectin and visceral fat in infants. Finally, anthropometric measures during the follow-up had about 5.3–6.0 % missing data in our study. However, analyses after multiple imputation of the missing values yielded similar findings and thus our findings were not biased by the missing data.

## Conclusions

Cord blood adiponectin is a determinant of fetal size at birth and weight gain during the first year of life. Maternal serum adiponectin during pregnancy may predict postnatal height growth.

## Abbreviations

BMI, body mass index; GEE, Generalized Estimating Equation; WHO, World Health Organization

## Additional file

Additional file 1: Table S1. The anthropometric measures at 3, 6 and 12 months of the infants. **Figure S1.** The Mean (SD) of weight-, height-, and BMI-for-age z scores at 0, 3, 6, 12 months of age. (DOC 190 kb)
