# Supplementary material for: Maternal and cord blood adiponectin levels in relation to post-natal body size in infants in the first year of life: a prospective study
Source: BMC Pregnancy Childbirth. 2016 Jul 27;16:189. doi: 10.1186/s12884-016-0978-9 (PMC4962456; doi:10.1186/s12884-016-0978-9)
Supplement: Additional file 1: Table S1. — The anthropometric measures at 3, 6 and 12 months of the infants. Figure S1. The Mean (SD) of weight-, height-, and BMI-for-age z scores at 0, 3, 6, 12 months of age. (DOC 190 kb) [file 12884_2016_978_MOESM1_ESM.doc]

Table S1The anthropometric measures at 3, 6 and 12 months of the infants.

| Variables | N | Median  ( interquartile range) |
| --- | --- | --- |
| Weight (kg) |  |  |
| 3 months | 405 | 6.5 (0.81) |
| 6 months | 405 | 7.8 (0.90) |
| 12 months | 408 | 9.8 (1.00) |
| Height (cm) |  |  |
| 3 months | 405 | 61.5 (3.0) |
| 6 months | 405 | 67.8 (2.8) |
| 12 months | 408 | 76.0 (2.2) |
| BMI (kg/cm^2^) |  |  |
| 3 months | 405 | 17.0 (1.2) |
| 6 months | 405 | 17.4 (1.5) |
| 12 months | 408 | 16.9 (1.3) |


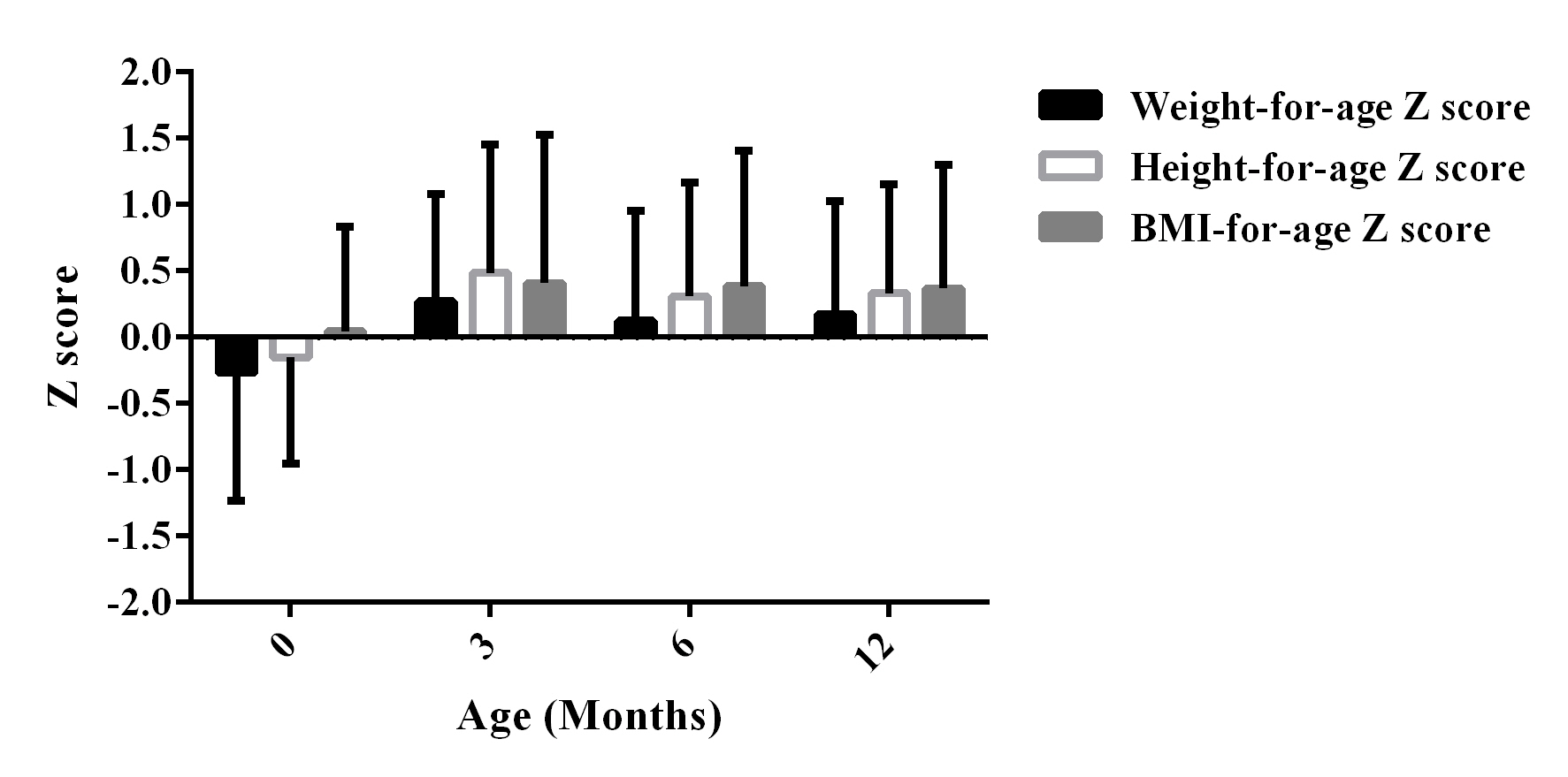


Fig S1 The Mean (SD) of weight-, height-, and BMI-for-age z scores at 0, 3, 6, 12 months of age.
